# Psychometric validation of the Spanish version of the Expanded Prostate Cancer Index Composite-26

**DOI:** 10.1007/s00345-023-04691-7

**Published:** 2023-11-10

**Authors:** Víctor Zamora, Olatz Garin, José Francisco Suárez, Josep Jové, Manuel Castells, Ferran Ferrer, Cristina Gutiérrez, Ferran Guedea, Ana Boladeras, Lluis Fumadó, Alvar Roselló, Jorge Pastor, Pilar Samper, Àngels Pont, Montse Ferrer

**Affiliations:** 1 Health Services Research Group, Hospital del Mar Research Institute, Barcelona Biomedical Research Park, Office 144. Doctor Aiguader, 88, 08003 Barcelona, Spain; 2 Universitat Autònoma de Barcelona (UAB), Bellaterra, Spain; 3 CIBER en Epidemiología y Salud Pública, CIBERESP, Madrid, Spain; 4 Universitat Pompeu Fabra, Barcelona, Spain; 5 Urology Department, Hospital Universitari de Bellvitge, L’Hospitalet de Llobregat, Spain; 6 Radiation Oncology Department, Institut Català d’Oncologia, Badalona, Spain; 7 Radiation Oncology Department, Institut Català d’Oncologia, L’Hospitalet de Llobregat, Spain; 8 Urology Department, Hospital del Mar, Barcelona, Spain; 9 Radiation Oncology Department, Institut Català d’Oncologia, Girona, Spain; 10 Radiation Oncology Department, ASCIRES GRUPO BIOMÉDICO, Valencia, Spain; 11 Radiation Oncology Department, Hospital Universitario Rey Juan Carlos, Móstoles, Spain

**Keywords:** EPIC-26, Expanded Prostate Cancer Index Composite, Patient-reported outcome measures, PROMs, Prostate cancer, Psychometric validation

## Abstract

**Purpose:**

To assess the validity, reliability, and responsiveness of the Spanish version of the Expanded Prostate cancer Index Composite (EPIC) with 26 items.

**Methods:**

Multicentric longitudinal study of patients diagnosed with localized or locally advanced prostate cancer (any T, any N, M0) treated with active surveillance, surgery, external radiotherapy, or brachytherapy. The EPIC-50 was administered initially to the cohort (*n* = 324 patients), until it was replaced in November 2019 by the EPIC-26 (*n* = 543), in both groups before treatment and 12 months after. We assessed confirmatory factor analysis (CFA), reliability with Cronbach’s alpha coefficient, criterion validity with the intraclass correlation coefficient (ICC), and responsiveness by testing a priori hypotheses on deterioration effect size (ES).

**Results:**

The CFA confirmed the five-domain structure of the EPIC-26 proposed by the original instrument (comparative fit index = 0.95). The agreement between EPIC-50 (gold standard) and EPIC-26 domains was excellent (ICC > 0.90). Cronbach’s alpha was > 0.7 in almost all domains, and the floor effect was near zero, although ceiling effect was higher than 50% in urinary incontinence and bowel domains. Hypothesized changes between before and 12 months after treatment were confirmed: ES > 0.8 in both urinary incontinence and sexual domains among patients who underwent surgery; and ES ranging 0.44–0.48 for bowel and sexual domains in patients treated with external radiotherapy.

**Conclusion:**

The Spanish version of the EPIC-26 has demonstrated adequate metric properties, similar to those of the original version, with acceptable goodness-of-fit indices, good criterion validity, reliability, and responsiveness to detect changes after radical prostatectomy or external radiotherapy.

**Supplementary Information:**

The online version contains supplementary material available at 10.1007/s00345-023-04691-7.

## Background

Prostate cancer is the most frequently diagnosed non-cutaneous cancer and the fifth cause of death among men in Europe and Spain [1]. Currently, most prostate cancer patients are diagnosed in localized stages [2] and will probably be long-term survivors [3]. Even so, these men may suffer relevant treatment and disease-related side-effects [4, 5], and thus, Patient-Reported Outcome Measures (PROMs) have become relevant endpoints that should be gathered from patients with localized or advanced prostate cancer [6].

The most established disease-specific PROM for these patients is the Expanded Prostate cancer Index Composite (EPIC), one of the instruments with the best properties for evaluating prostate cancer care [7, 8]. It is composed by 50 items, and was developed to expand the scope of the original 20-item University of California, Los Angeles Prostate Cancer Index (UCLA-PCI), with additional items that assess irritating urinary symptoms and the impacts of hormonal therapy [9].

In 2010, a new abbreviated version with 26 items (EPIC-26) was developed by assessing items for elimination through an iterative process. Each domain was correlated with the corresponding scores from the full EPIC-50, and this process was repeated until all psychometric properties reached acceptable levels [9]. The EPIC-26 has been included in the standard set of outcomes recommended by the International Consortium for Health Outcomes Measurement (ICHOM) [6].

Psychometric evaluations of EPIC-26 are available for several country versions (Norway, China, Germany, Italy, and Canada [10–15]). However, this assessment has not been performed for the Spanish version of the EPIC-26 [16], which was constructed by selecting the corresponding items from the Spanish version of EPIC-50 [17]. The aim of this study is to assess the metric properties of the Spanish version of EPIC-26 in a sample of Spanish men with prostate cancer, in terms of validity, reliability, and sensitivity to change.

## Methods

### Study design

Data came from a multicentric observational cohort study of Spanish patients diagnosed with localized or locally advanced prostate cancer (any T, any N, M0), recruited between 2017 and 2021 from 17 Spanish hospitals which are part of the ongoing international True North Global Registry [18].

Participants’ demographic and clinical characteristics were collected by physicians, and PROMs were administered centrally through telephone interviews before and 12 months after primary treatment, or after the beginning of active surveillance. The ethics review boards of all participating Spanish hospitals approved the study, and written informed consent was requested from patients (Research Ethics Committee (CRE) at Parc de Salut Mar: TrueNTH_PCO).

### Measures

The EPIC-50 was initially administered to the cohort patients until November 2019, when it was replaced by the EPIC-26 due to its lower burden. Both EPIC-50 [19] and EPIC-26 [10] measure five domains: urinary incontinence (both with 4 items), urinary irritative/obstructive symptoms (with 7 and 4 items, respectively), sexual (13 and 6 items), bowel (14 and 6 items), and hormonal (11 and 5 items). Both versions have response options with 4-, 5-, or 6-level Likert scales, and these are linearly transformed to a scale of 0–100, where higher scores indicate better outcomes. Items are grouped into summary scores for sexual, bowel, and hormonal domains, and into two urinary domains: incontinence and irritative/obstructive symptoms [10].

The Spanish version of the EPIC-50 was obtained through a standard linguistic adaptation process described elsewhere [17]. Briefly, two forward and backward translations were performed to obtain a preliminary Spanish version, and cognitive debriefing interviews were carried out to ten patients with prostate cancer. These patients were asked to respond to this preliminary Spanish version of EPIC-50, to check understandability, interpretation, and cultural relevance of the content, as well as to identify alternative wording if necessary. Two items (bowel frequency and breast problems) were slightly modified according to the patients’ comments, without penalizing semantic equivalence, thus achieving a definitive Spanish version that is conceptually equivalent to the original EPIC-50 [17]. The Spanish version of EPIC-26 was derived from the items of the Spanish version of EPIC-50.

### Statistical analysis

Differences at baseline between patients completing EPIC-50 and EPIC-26 versions were tested using either the Chi-square test or the unpaired Student’s *t* test. The observed range of EPIC scores at 12 months after treatment, central tendency and dispersion statistics, and ceiling and floor effects were calculated. Reliability was assessed through Cronbach’s alpha [20] as an indicator of internal consistency.

Construct validity was assessed by performing a confirmatory factor analysis (CFA) to examine the five-domain structure defined in the original EPIC-26, applying the evaluation at 12 months after treatment. The Root-Mean-Square Error of Approximation (RMSEA), and the relative fit of the specified model was assessed with the Comparative Fit Index (CFI) and Tucker-Lewis Index (TLI). For these statistics, RMSEA values below than 0.08 and CFI and TLI values above 0.90 indicate an acceptable fit [21].

Scatter plots between EPIC-26 and EPIC-50 (gold standard) were constructed, and Intraclass Correlation Coefficients (ICC) were calculated to assess the criterion validity in the subsample of patients who answered the EPIC-50. The domain scores of EPIC-26 were calculated by selecting its constituent items from the EPIC-50. The agreement between versions was hypothesized to be excellent (ICC > 0.90) [22].

Differences in EPIC scores between pretreatment and 12 months after in patients undergoing radical prostatectomy and external radiotherapy were tested to assess the sensitivity to change through the paired Student’s t test. Cohen’s effect sizes (ES) were calculated as the difference between the means at each time-point of EPIC scores divided by the pooled SD, considered as small (ES = 0.2), moderate (ES = 0.5), or large changes (ES = 0.8) [23]. Based on scientific evidence, we hypothesized large deterioration in urinary incontinence and sexual domains after radical prostatectomy [4, 24], and moderate deteriorations in bowel and sexual domains after external radiotherapy [25]. All analyses were performed using R version 4.2.2., and the CFA was constructed with the ‘lavaan’ package.

## Results

Characteristics of patients and the distribution of EPIC scores at 12 months after treatment are summarized in Table 1, separately for those who responded to EPIC-26 (*n* = 543) and to EPIC-50 (*n* = 324). In both groups, most participants were categorized at intermediate D’Amico tumoral risk (≈ 40%), and external radiotherapy was the predominant treatment, followed by radical prostatectomy, brachytherapy as monotherapy, and active surveillance. Four patients from the radical prostatectomy group received salvage radiotherapy, and 64 from external radiotherapy group received a boost of brachytherapy. The distribution of the EPIC-26 and EPIC-50 scores at 12 months after treatment shows that the observed range is very similar in all domains to the theoretical range (from 0 to 100), except in the sexual domain. The floor effect was lower than 2% in all domains, but the ceiling effect (percentage of patients with the best outcome) was high in all domains, except for sexual. It was greater than 50% in urinary incontinence and bowel in both versions. Cronbach’s alpha was greater than 0.7 in all domains, except in the urinary irritative/obstructive domain, which was 0.67 for EPIC-26 and 0.63 for EPIC-50. Distribution of EPIC-26 items at 12 months after treatment (absolute and relative frequencies) is presented in Supplementary Table 1.

**Table 1:** Characteristics and treatments of patients according to EPIC version administered. Distribution of the EPIC domains’ scores at 12 months after treatment

	Patients with EPIC-26 (*n* = 534)	Patients with EPIC-50 (*n* = 324)	*p* value*
**Characteristics**			
*Age at recruitment, years, mean (SD)*	68.1 (8.0)	67.9 (8.0)	0.652
*Tumoral stage, n (%)*			
Tx	8 (1.5%)	1 (0.3%)	0.346
T0	1 (0.2%)	1 (0.3%)	
T1	250 (46.0%)	162 (50.2%)	
T2	184 (33.9%)	102 (31.6%)	
T3	97 (17.9%)	57 (17.6%)	
T4	3 (0.6%)	0 (0.0%)	
*PSA, mean (SD)*	11.1 (13.8)	9.4 (9.0)	0.049
*Gleason score, mean (SD)*	7.1 (0.9)	6.8 (1.0)	< 0.001
*D’Amico tumoral risk, n (%)*			
Low	94 (17.4%)	102 (31.6%)	< 0.001
Intermediate	249 (46.1%)	128 (39.6%)	
High	197 (36.5%)	93 (28.8%)	
*Initial primary treatment*			
External radiotherapy, n (%)	282 (51.9%)	181 (55.9%)	< 0.001
Radical prostatectomy, n (%)	185 (34.1%)	83 (25.6%)	
Brachytherapy as monotherapy, n (%)	38 (7.0%)	44 (13.6%)	
Active surveillance, n (%)	29 (5.3%)	3 (0.9%)	
Focal brachytherapy, n (%)	9 (1.7%)	13 (4.0%)	
**Distribution of the EPIC scores**			
*Urinary incontinence*			
Observed range	0–100	0–100	
Floor and ceiling effect (%)	0.9%; 54.1%	1.9%; 62.7%	
Mean (SD)	77.9 (28.2)	81.5 (27.5)	
Cronbach’s alpha	0.90	0.91	
*Urinary irritative/obstructive*			
Observed range	0–100	7.1–100	
Floor and ceiling effect (%)	0.7%; 42.5%	0.0%; 36.4%	
Mean (SD)	80.6 (22.5)	84.5 (17.7)	
Cronbach’s alpha	0.67	0.63	
*Bowel*			
Observed range	0–100	28.6–100	
Floor and ceiling effect (%)	0.4%; 66.1%	0.0%; 61.7%	
Mean (SD)	88.1 (21.2)	92.0 (14.1)	
Cronbach’s alpha	0.84	0.87	
*Sexual*			
Observed range	0–87.5	0–90.4	
Floor and ceiling effect (%)	1.8%; 0.0%	0.3%; 0.0%	
Mean (SD)	34.8 (27.4)	43.6 (19.9)	
Cronbach’s alpha	0.86	0.88	
*Hormonal*			
Observed range	0–100	4.6–100	
Floor and ceiling effect (%)	1.3%; 38.9%	0.0%; 29.9%	
Mean (SD)	74.2 (28.0)	82.2 (19.7)	
Cronbach’s alpha	0.78	0.84	

*p-values were estimated with Chi-square test or unpaired Student’s *t* test

**Fig. 1:**
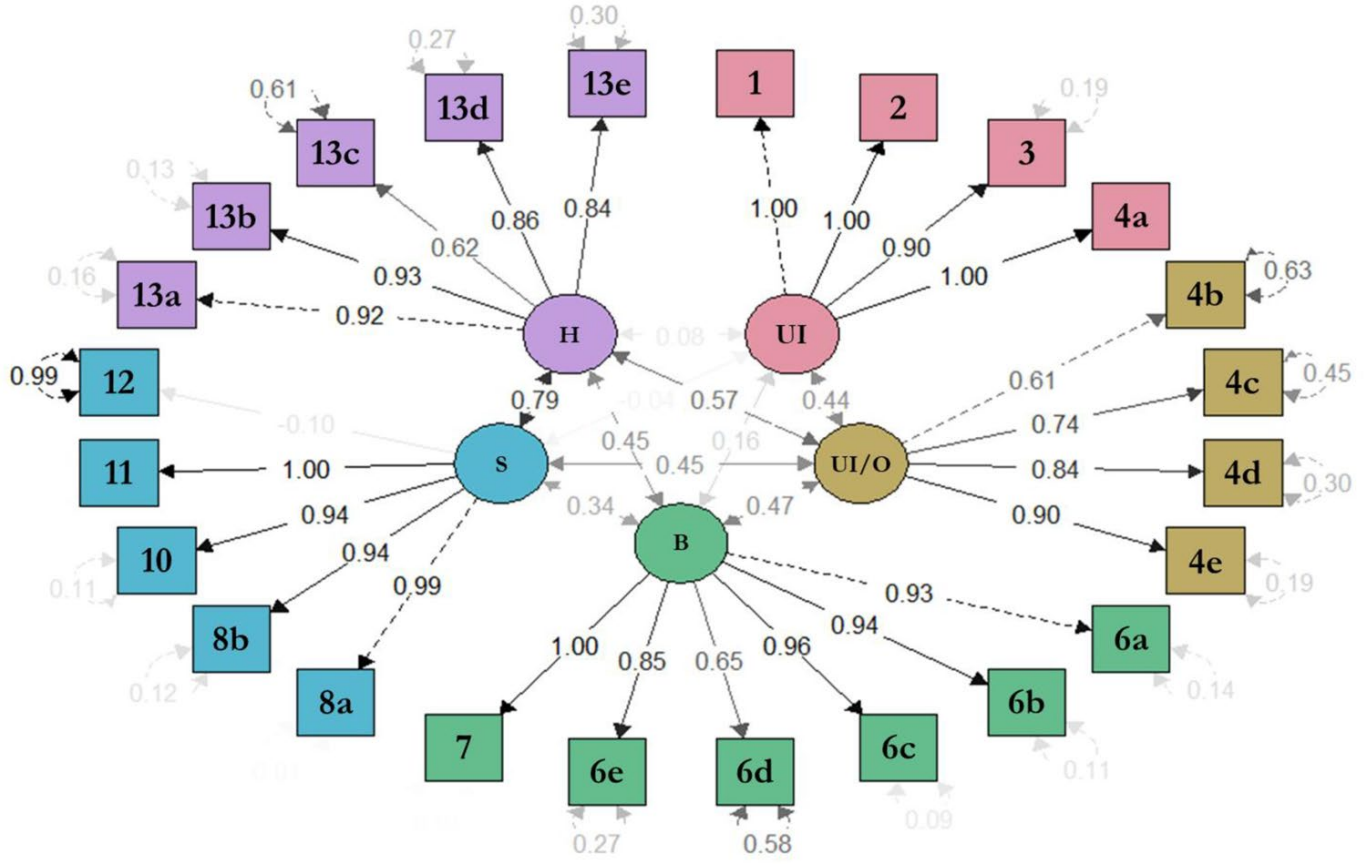
Confirmatory factor analysis of the 5-factor structure of the EPIC-26 Spanish version. CFI = 0.99; and TLI = 0.99; RMSEA = 0.066; *UI* urinary incontinence, *UI/O* urinary irritative/obstructive, *B* bowel, *S* sexual, *H* hormonal. Numbers inside the arrows represent the correlation between domains, between item and domain, and within the item itself. The intensity of the gray shadowing in the numbers represents the intensity of the correlation, darker numbers being higher correlations

Results of the confirmatory factor analysis of EPIC-26 with a five-domain structure is represented in Fig. 1, showing acceptable goodness-of-fit indexes: RMSEA = 0.066; CFI and TLI > 0.95. The inter-domains covariances ranged from 0.16 (between urinary incontinence and bowel) to 0.79 (between hormonal and sexual). Most of the standardized regression weights between each item and their domain are greater than 0.6. For instance, the weights between items ‘4b-dysuria’, ‘4c-hematuria’, ‘4d-weak stream’, and ‘4e-frequency’ in the urinary irritative/obstructive domain is 0.61, 0.74, 0.84, and 0.90, respectively. The residuals were lower than 0.5 in all items (the lower the values, the better), except for four items: ‘4b-dysuria’, ‘6d-bloody stools’, ‘12-overall sexual problem’, and ‘13c-depression’.

Supplementary Fig. 1 represents the scatter plots constructed from the subsample of patients who answered the EPIC-50, and the agreement between their scores and those scores obtained by selecting the items that constitute the EPIC-26. The agreement between them was excellent for all domains, with ICCs higher than 0.95.

Table 2 shows the results of the EPIC-26 and the EPIC-50 for the subsample of patients that underwent radical prostatectomy (*n* = 95 and *n* = 79, respectively) or external radiotherapy (*n* = 115 and *n* = 170, respectively). The patients who underwent radical prostatectomy presented statistically significant changes in urinary incontinence, bowel, and sexual domains, which were of large magnitude (ES > 0.8) in both versions. In those patients treated with external radiotherapy, changes in bowel, sexual, and hormonal domains were statistically significant, and the ES ranged from 0.44 to 0.69 with EPIC-26, and from 0.3 to 1.09 with EPIC-50.

**Table 2:** Sensitivity to change of the EPIC-50 and the EPIC-26 by treatment group

Domains and items	Mean (SD) at baseline (T0)		Mean (SD) at 12 months (T1)		Mean difference (SD) (T1 – T0)		Cohen’s Effect Size	
	EPIC-26	EPIC-50	EPIC-26	EPIC-50	EPIC-26	EPIC-50	EPIC-26	EPIC-50
*Radical prostatectomy*	*n* = 95	*n* = 79	*n* = 95	*n* = 79	*n* = 95	*n* = 79	*n* = 95	*n* = 79
Urinary incontinence	93.9 (14.9)	94.3 (16.0)	60.3 (32.3)	59.7 (31.9)	− 33.6 (0.0)*	− 34.6 (34.7)*	2.25	2.16
Urinary irritative/obstructive	79.9 (19.8)	89.6 (15.8)	80.6 (21.5)	85.5 (20.4)	0.7 (35.0)	− 4.1 (22.2)	0.04	0.26
Bowel	96.5 (8.6)	98.4 (4.3)	89.4 (21.8)	95.0 (11.9)	− 7.1 (24.5)*	− 3.4 (12.0)*	0.83	0.78
Sexual	68.0 (28.4)	71.6 (17.1)	40.9 (28.9)	53.4 (25.1)	− 27.1 (22.7)*	− 18.3 (24.6)*	0.95	1.07
Hormonal	85.3 (13.1)	88.9 (11.4)	81.6 (26.9)	89.2 (15.8)	− 3.8 (32.7)	0.3 (14.9)	0.29	0.03
*External radiotherapy*	*n* = 115	*n* = 170	*n* = 115	*n* = 170	*n* = 115	*n* = 170	*n* = 115	*n* = 170
Urinary incontinence	86.9 (20.6)	86.1 (21.5)	85.0 (23.3)	87.7 (21.4)	− 1.9 (25.6)	1.7 (21.2)	0.09	0.08
Urinary irritative/obstructive	76.1 (19.8)	85.8 (14.9)	76.9 (23.7)	83.2 (17.2)	0.8 (0.0)	− 2.6 (16.9)	0.04	0.17
Bowel	93.5 (13.6)	97.5 (6.2)	87.0 (21.9)	90.7 (15.1)	− 6.5 (21.1)*	− 6.7 (15.4)*	0.48	1.09
Sexual	34.4 (28.9)	41.9 (19.2)	21.7 (17.5)	36.2 (14.2)	− 12.7 (25.9)*	− 5.8 (16.1)*	0.44	0.30
Hormonal	77.0 (21.8)	81.1 (15.2)	61.9 (31.2)	75.1 (20.8)	− 15.1 (24.1)*	− 6.0 (19.6)*	0.69	0.40

*p value was estimated with paired Student’s *t* test for each subsample and EPIC version

## Discussion

The Spanish version of the EPIC-26 has demonstrated adequate metric properties, similar to those of the original version, with acceptable goodness-of-fit indexes in the confirmatory factor analysis of the five-domain structure and good criterion validity compared to the Spanish EPIC-50. It presented a very low floor effect (< 2%), good reliability in almost all domains (Cronbach’s alpha > 0.7), and responsiveness to detect change after radical prostatectomy or external radiotherapy. However, the ceiling effect was high, especially in urinary incontinence and bowel domains (> 50%).

The Spanish version of the EPIC-26 demonstrated an acceptable fit to the five-domain structure proposed by the original instrument [10]. These results support the construct validity of calculating separate scores for urinary incontinence, urinary irritative/obstructive, bowel, sexual, and hormonal symptoms, similarly to the original study [10] and the German version [13]. The Norwegian [11] and the Canadian version [15] found that a six-domain structure model fits better, splitting up the hormonal domain into two subdomains. Consistently with results of the CFA obtained in the Norwegian [11], the German [13], and the Canadian version [15], we also found it difficult to make the model converge, which was solved by dichotomization of items (no problems vs problems), and the exclusion of the item ‘9-Erection not firm’.

Criterion validity results showed an excellent agreement (ICC > 0.90 [22]) between all EPIC-26 domains and those from the original EPIC-50. A similar evaluation has only been performed in the original American version study [10]. Despite estimating different validity parameters, the results for criterion validity of EPIC-26 compared to EPIC-50 domains were almost identical: the correlation r ranged from 0.96 to 0.97 in the original version [10], and the ICC ranged from 0.96 to 0.98 in our study. This indicates that regardless of the EPIC version a patient completed, the domain scores obtained would be practically the same. Hence, considering its low burden of administration, the abbreviated version with 26 items is more appropriate.

The Spanish version of the EPIC-26 presented a floor effect near zero, in line with those observed in other studies of EPIC-26 versions [11, 15], and meets the recommended quality criteria for floor effect (lower than 15%) [26]. In contrast, bowel and urinary incontinence domains exhibited high ceiling effects (% of patients with the best outcomes), similarly to the studies of the American [10], the Norwegian [11], and the Canadian versions [15]. Nonetheless, these percentages in our study were higher (66.1% and 54.1%, respectively) than in the previous publications. For instance, in the bowel domain: 34% in the American version, and close to 62% in other versions [11, 15]. Considering these high percentages of patients reporting no problems in bowel items, as well as in urinary incontinence, the evaluation of the EPIC-26 validity in those patients with slight or mild symptoms in these domains merits further research. It is important to consider that the EPIC-26 was developed to measure the impact of a broad spectrum of treatments differing in side-effects [9, 10], which explains the variation of the ceiling effect across different samples according to treatments applied: for instance, urinary incontinence is common after surgery, and bowel discomfort after radiotherapy.

Reliability results achieved the acceptable threshold for internal consistency (Cronbach’s alpha > 0.7) [20] in most EPIC-26 domains. They were very similar to those obtained by the EPIC-50, indicating that the EPIC-26 contains enough items to measure domains without internal consistency penalization. Similarly to the Chinese [12], German [13], and Italian version [14], the urinary irritative/obstructive domain is the only one that showed poor internal consistency in our study (Cronbach’s alpha = 0.67). This may be explained by the item ‘4c-Hematuria’, with an extreme percentage of patients reporting no problems (98.5%). In fact, Cronbach’s alpha of this domain was 0.71 when excluding this item (data not shown). This is in line with the Italian version study [14], which highlights the need of further research to identify more reliable new urinary irritative/obstructive items. However, the most recently developed prostate cancer-specific PROM, the European Organization for Research and Treatment of Cancer Quality of Life Questionnaire-Prostate (EORTC QLQ-PR25), does not measure urinary irritative/obstructive symptoms [27].

The Spanish version of the EPIC-26 is able to detect changes between before and 12 months after treatment as hypothesized a priori, according to clinically known side-effects of surgery or external radiotherapy for prostate cancer [4, 24, 25]. On the one hand, patients who underwent external radiotherapy presented statistically significant deteriorations of moderate magnitude in bowel, sexual, and hormonal domains (ES ranging from 0.44 to 0.69). On the other hand, patients who underwent radical prostatectomy presented statistically significant deteriorations of large magnitude in urinary incontinence (ES = 2.25) and sexual domains (ES = 0.95). To the best of our knowledge, our study is the first one that assesses EPIC-26 responsiveness for surgery and external radiotherapy, since this property has not been evaluated in the original American version nor in other studies of EPIC-26 versions. Although the German study [13] also evaluated responsiveness, they considered the whole sample (84% of patients underwent radical prostatectomy and 16% had other treatments), even though side-effects differ according to the treatments applied. Our results of patients who underwent radical prostatectomy are in line with the deterioration of large magnitude obtained in the German study (ES = 1.22 for urinary incontinence and ES = 1.15 for sexual domains) [13].

The main limitation of this study is that it includes patients with localized or locally advanced prostate cancer, mostly treated with external radiotherapy or radical prostatectomy, which limits the generalizability of the results to patients with metastatic disease or to patients who underwent other treatments. However, the sample is heterogeneous enough to represent most tumoral stages, as well as the most currently established treatments for non-metastatic disease. Furthermore, test–retest reproducibility could not be studied, since the time elapsed between administrations was too long to assume the patients’ stability. Nevertheless, good results of internal consistency support the reliability of the EPIC-26.

In conclusion, this study represents the first evaluation of the psychometric properties of the Spanish version of the EPIC-26, which can be considered a reliable and valid instrument to analyze the impact of different treatments in patients with localized or locally advanced prostate cancer. The good responsiveness of EPIC-26 to detect changes after treatment supports its usefulness for the clinical decision-making process in these patients. In addition, the low burden of administration makes the EPIC-26 a practical tool for its routine use in clinical practice and in international multicentric studies, and it could facilitate benchmarking among registries.

### Supplementary Information

Below is the link to the electronic supplementary material.

Supplementary file1 (PDF 95 KB)

Supplementary file2 (PDF 196 KB)

## Data Availability

The datasets generated and analyzed during the current study are available from the corresponding authors only under a data-sharing agreement request. There are no restrictions on the use of published data after project completion, but we expect users to follow standard scientific citation guidelines and they will need to acknowledge the source of the data in any resulting publication.
