# Supplementary material for: Psychometric validation of the Spanish version of the Expanded Prostate Cancer Index Composite-26
Source: World J Urol. 2023 Nov 10;41(12):3511–8. doi: 10.1007/s00345-023-04691-7 (PMC10693511; doi:10.1007/s00345-023-04691-7)
Supplement: Supplementary file 1 — Supplementary file1 (PDF 95 KB) [file 345_2023_4691_MOESM1_ESM.pdf]

Psychometric validation of the Spanish version of the Expanded Prostate cancer Index Composite-26

World Journal of Urology

Víctor Zamora MPH[1,2,3], Olatz Garin PhD[1,3,4]*, José Francisco Suárez MD[5], Josep Jové MD[6], Manuel Castells MD[5], Ferran Ferrer MD[7], Cristina Gutiérrez PhD[7], Ferran Guedea PhD[7], Ana Boladeras MD[7], Lluis Fumadó PhD[8], Alvar Roselló MD[9], Jorge Pastor MD[10], Pilar Samper PhD[11], Àngels Pont MSc[1,3], Montse Ferrer PhD[1,3,4]*

1 Health Services Research Group, Hospital del Mar Research Institute, Barcelona, Spain.

2 Universitat Autònoma de Barcelona (UAB), Bellaterra, Spain.

3 CIBER en Epidemiología y Salud Pública, CIBERESP, Spain.

4 Universitat Pompeu Fabra, Barcelona, Spain.

5 Urology Department, Hospital Universitari de Bellvitge, L'Hospitalet de Llobregat, Spain.

6 Radiation Oncology Department, Institut Català d'Oncologia, Badalona, Spain.

7 Radiation Oncology Department, Institut Català d'Oncologia, L'Hospitalet de Llobregat, Spain.

8 Urology Department, Hospital del Mar, Barcelona, Spain.

9 Radiation Oncology Department, Institut Català d'Oncologia, Girona, Spain.

10 Radiation Oncology Department, ASCIRES GRUPO BIOMÉDICO, Valencia, Spain.

11 Radiation Oncology Department, Hospital Universitario Rey Juan Carlos, Móstoles, Spain

Contact information for corresponding authors

*Olatz Garin and Montse Ferrer, PhD,

e-mail address: ogarin@imim.es; mferrer@imim.es

Supplementary Table 1. Absolute and relative frequencies of EPIC-26 response options at 12 months after treatment (n=543).

EPIC-26	Response options, n (%)				
Items, n (%)	The Best				The Worst
Urinary incontinence					
1. Leaking	311 (57.3%)	95 (17.5%)	54 (9.9%)	31 (5.7%)	52 (9.6%)
2. Dribbling	295 (54.3%)	148 (27.3%)	95 (17.5%)	5 (0.9%)	---
3. Pad use*	455 (83.8%)	57 (10.5%)	20 (3.7%)	11 (2.0%)	---
4a. Leaking problem*	294 (54.1%)	5 (0.9%)	57 (10.5%)	95 (17.5%)	92 (16.9%)
Urinary Irritative/Obstructive					
4b. Dysuria*	462 (85.1%)	1 (0.2%)	32 (5.9%)	30 (5.5%)	18 (3.3%)
4c. Hematuria*	535 (98.5%)	0 (0.0%)	0 (0.0%)	0 (0.0%)	8 (1.5%)
4d. Weak stream*	362 (66.7%)	2 (0.4%)	43 (7.9%)	70 (12.9%)	66 (12.2%)
4e. Frequency*	267 (49.2%)	1 (0.2%)	53 (9.8%)	127 (23.4%)	95 (17.5%)
5. Overall urinary problem*	194 (35.7%)	1 (0.2%)	87 (16.0%)	148 (27.3%)	113 (20.8%)
Bowel					
6a. Urgency*	486 (89.5%)	0 (0.0%)	11 (2.0%)	27 (5.0%)	19 (3.5%)
6b. Frequency*	462 (85.1%)	0 (0.0%)	13 (2.4%)	42 (7.7%)	26 (4.8%)
6c. Fecal incontinence*	480 (88.4%)	0 (0.0%)	2 (0.4%)	20 (3.7%)	41 (7.6%)
6d. Bloody stools*	492 (90.6%)	0 (0.0%)	15 (2.8%)	19 (3.5%)	17 (3.1%)
6e. Rectal pain*	474 (87.3%)	0 (0.0%)	13 (2.4%)	39 (7.2%)	17 (3.1%)
7. Overall bowel problem*	372 (68.5%)	0 (0.0%)	40 (7.4%)	79 (14.5%)	52 (9.6%)
Sexual					
8a. Poor erections	0 (0.0%)	99 (18.2%)	59 (10.9%)	83 (15.3%)	302 (55.6%)
8b. Difficulty with orgasm	2 (0.4%)	128 (23.6%)	74 (13.6%)	44 (8.1%)	295 (54.3%)
9. Erection not firm	123 (22.7%)	87 (16.0%)	64 (11.8%)	269 (49.5%)	---
10. Erections frequency	86 (15.8%)	47 (8.7%)	61 (11.2%)	24 (4.4%)	325 (59.9%)
11. Poor sexual function	0 (0.0%)	89 (16.4%)	63 (11.6%)	58 (10.7%)	333 (61.3%)
12. Overall sexual problem*	355 (65.4%)	0 (0.0%)	31 (5.7%)	86 (15.8%)	71 (13.1%)
Hormonal					
13a. Hot flashes*	384 (70.7%)	0 (0.0%)	20 (3.7%)	46 (8.5%)	93 (17.1%)
13b. Breast problems*	437 (80.5%)	4 (0.7%)	54 (9.9%)	38 (7.0%)	10 (1.8%)
13c. Depression*	312 (57.5%)	1 (0.2%)	29 (5.3%)	82 (15.1%)	119 (21.9%)
13d. Lack of energy*	307 (56.5%)	0 (0.0%)	25 (4.6%)	78 (14.4%)	133 (24.5%)
13e. Weight change*	415 (76.4%)	2 (0.4%)	31 (5.7%)	51 (9.4%)	44 (8.1%)

**The direction of these items is opposite (lower scores mean better outcomes*
