# Supplementary material for: Psychometric validation of the Spanish version of the Expanded Prostate Cancer Index Composite-26
Source: World J Urol. 2023 Nov 10;41(12):3511–8. doi: 10.1007/s00345-023-04691-7 (PMC10693511; doi:10.1007/s00345-023-04691-7)
Supplement: Supplementary file 2 — Supplementary file2 (PDF 196 KB) [file 345_2023_4691_MOESM2_ESM.pdf]

Psychometric validation of the Spanish version of the Expanded Prostate cancer Index Composite-26

World Journal of Urology

Víctor Zamora MPH[1,2,3], Olatz Garin PhD[1,3,4]*, José Francisco Suárez MD[5], Josep Jové MD[6], Manuel Castells MD[5], Ferran Ferrer MD[7], Cristina Gutiérrez PhD[7], Ferran Guedea PhD[7], Ana Boladeras MD[7], Lluis Fumadó PhD[8], Alvar Roselló MD[9], Jorge Pastor MD[10], Pilar Samper PhD[11], Àngels Pont MSc[1,3], Montse Ferrer PhD[1,3,4]*

1 Health Services Research Group, Hospital del Mar Research Institute, Barcelona, Spain.

2 Universitat Autònoma de Barcelona (UAB), Bellaterra, Spain.

3 CIBER en Epidemiología y Salud Pública, CIBERESP, Spain.

4 Universitat Pompeu Fabra, Barcelona, Spain.

5 Urology Department, Hospital Universitari de Bellvitge, L'Hospitalet de Llobregat, Spain.

6 Radiation Oncology Department, Institut Català d'Oncologia, Badalona, Spain.

7 Radiation Oncology Department, Institut Català d'Oncologia, L'Hospitalet de Llobregat, Spain.

8 Urology Department, Hospital del Mar, Barcelona, Spain.

9 Radiation Oncology Department, Institut Català d'Oncologia, Girona, Spain.

10 Radiation Oncology Department, ASCIRES GRUPO BIOMÉDICO, Valencia, Spain.

11 Radiation Oncology Department, Hospital Universitario Rey Juan Carlos, Móstoles, Spain

Contact information for corresponding authors

*Olatz Garin and Montse Ferrer, PhD,

e-mail address: ogarin@imim.es; mferrer@imim.es

Supplementary Figure 1. Assessment of criterion validity between each domain of the EPIC-50 and the same domain in EPIC-26 [Intraclass Correlation Coefficient (ICC)], in 324 patients recruited between January 2017 and November 2019.

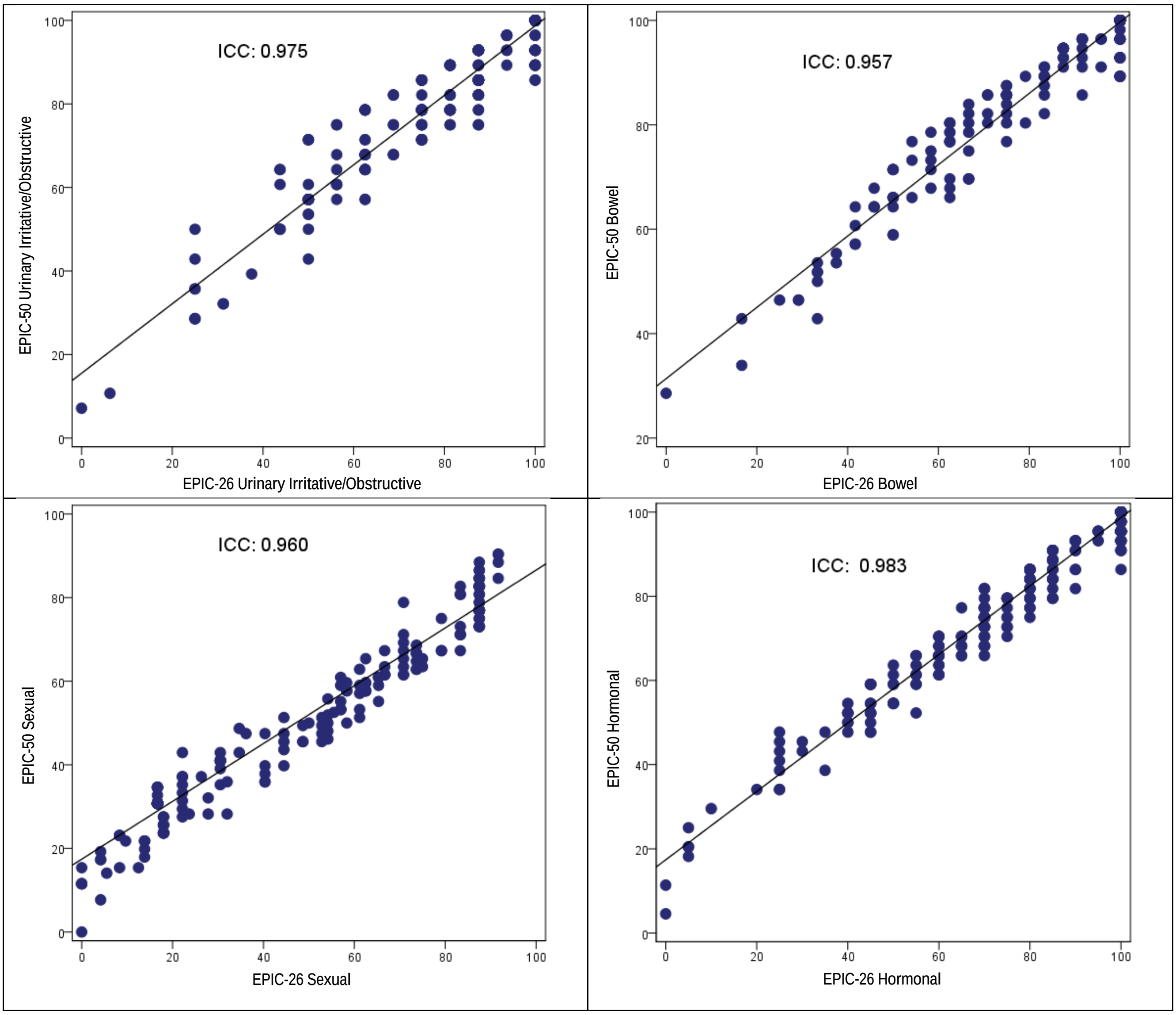

Footnote: *Urinary Incontinence was not represented because the four items from both EPIC versions are equal.*
